# Observational study of potential risk factors of immersion pulmonary edema in healthy divers: exercise intensity is the main contributor

**DOI:** 10.1186/s40798-017-0104-1

**Published:** 2017-10-03

**Authors:** A. Boussuges, K. Ayme, G. Chaumet, E. Albier, M. Borgnetta, O. Gavarry

**Affiliations:** 1 UMR MD2, Dysoxie-Suractivité, Aix-Marseille Université et Institut de Recherche Biomédicale des Armées (IRBA), Faculté de Médecine Nord, Marseille, France; 2 Altrabio, Lyon, SA France; 3 Institut National de Plongée Professionnelle, Port de la Pointe Rouge, Marseille, France; 4 Laboratoire HandiBio EA 4322, Université de Toulon, La Garde, France

**Keywords:** Diving medicine, Pulmonary edema, Ultrasound, Chest ultrasonography

## Abstract

**Background:**

The risk factors of pulmonary edema induced by diving in healthy subjects are not well known. The aim of the present study was to assess the parameters contributing to the increase in extravascular lung water after diving.

**Methods:**

This study was carried out in a professional diving institute. All divers participating in the teaching program from June 2012 to June 2014 were included in the study. Extravascular lung water was assessed using the detection of ultrasound lung comets (ULC) by chest ultrasonography. Clinical parameters and dive profiles were recorded using a questionnaire and a dive computer.

**Results:**

One-hundred six divers were investigated after 263 dives. They used an open-circuit umbilical supplying compressed gas diving apparatus in 202 cases and a self-contained underwater breathing apparatus in 61 cases. A generalized linear mixed model analysis was performed which demonstrated that the dive induced a significant increase in ULC score (incidence rate ratio: 3.16). It also identified that the predictive variable of increased extravascular lung water after the dive was the exercise intensity at depth (*z* = 3.99, *p* < 0.0001). The other parameters studied such as the water temperature, dive profile, hyperoxic exposure, or anthropometric data were not associated with the increase in extravascular lung water after the dive.

**Conclusions:**

In this study, the exercise intensity was the main contributor to the increase in extravascular lung water in healthy divers. To improve the prevention of immersion pulmonary edema, the exercise intensity experienced during the dive should thus be adapted to the aerobic fitness level of the divers.

## Key points

- This is the first prospective study investigating the factors implicated in the increase in extravascular lung water after diving.
- An important increase in extravascular lung water rarely occurs after a well-controlled dive.
- The main contributor to the increase in extravascular lung water in a healthy diver is the exercise intensity.

## Background

Pulmonary edema (PE) induced by SCUBA diving has been described in 1981 by Wilmshurst et al. [1]. Since this initial report, several cases of PE induced by various aquatic activities such as swimming, aquagym, breath-hold diving, and SCUBA diving have been reported [2–5]. The injuries may be under-reported, as the intensity of the clinical disorders may vary from minimal symptoms (transitory cough) to acute respiratory distress. Consequently, some divers may not apply for medical attention. However, diagnosis remains important because when recurrent injuries have been reported, the second episode of PE can be fatal [6]. In some cases, PE occurred in divers with cardiac disease [7–9]. Most frequently, the injury affected divers with an apparently normal heart [10]. A deeper understanding of the pathophysiology of the SCUBA diving-induced PE is important to better prevent the injury. Risk factors probably included environmental stressors and individual factors of susceptibility. Environmental stressors experienced during SCUBA diving such as water immersion, cold exposure, exercise intensity, hyperoxia, and decompression have been implicated in the pathogenesis [11]. Furthermore, the injury occurred more frequently in one sole diver among a group of divers performing the same dive profile, suggesting that some individual risk factors existed. In a recent review, Peacher et al. [12] reported that among recreational divers, risk factors of cardio-respiratory disorders are more frequent than initially estimated (from 44 to 72%). Other risk factors such as female gender [13, 14], advanced age [4], consumption of fish oil [14] or anti-platelet agent [2], hypertension [15], and obesity [12] have also been suggested.

**Fig. 1:**
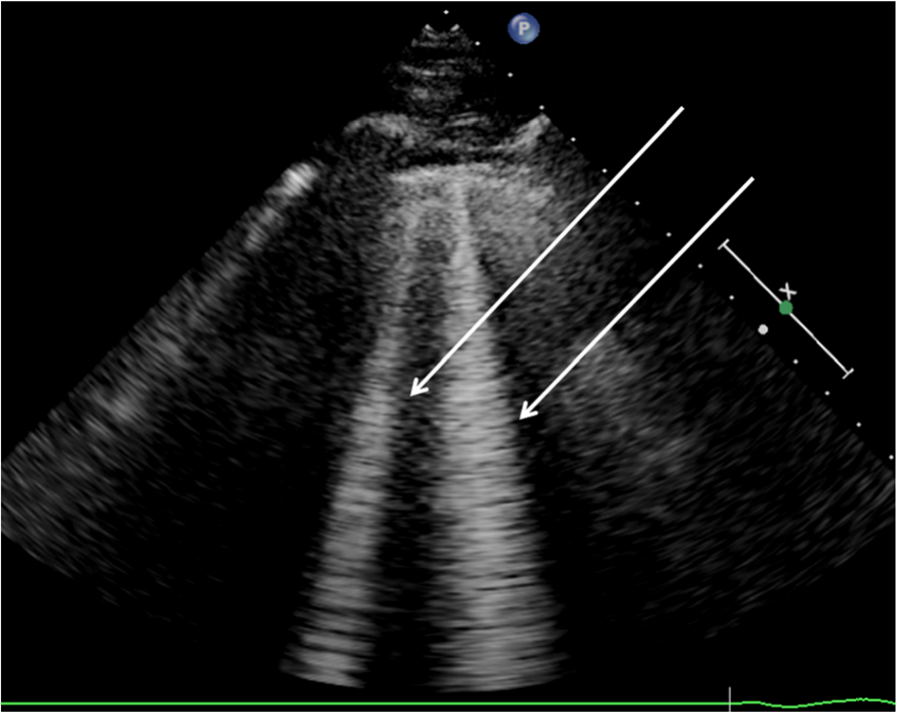
Two ultrasound lung comets (arrows) arising from the pleural line

For a better understanding of the pathogenesis of this injury, a prospective study is needed. Nevertheless, immersion pulmonary edema (IPE) is a rare event. The frequency of the disease has been assessed in an epidemiologic study performed on 1250 divers and swimmers. From this group, 1.1% presented symptoms related to IPE [4]. To undertake a prospective study, a procedure that can detect clinically silent PE would be advantageous. Chest ultrasonography is recognized as an interesting tool to visualize alveolo-interstitial syndrome using specific images called ULC (Ultrasound lung comets, Fig. 1) [16]. It has been used to quantify extravascular lung water (EVLW) in detecting minor PE in both climbing victims of mountain sickness [17] and athletes performing heavy exercise at sea level [18]. Chest ultrasonography has also been performed after apnea competitions demonstrating that breath-hold diving could increase the ULC score (ULCs) particularly in divers with clinical troubles [19, 20].

The present study was therefore designed to assess the environmental and individual factors promoting an increase in EVLW in a large population of professional divers with no medical history of cardio-respiratory disease. We hypothesized that risk factors suggested in previous studies such as cold temperature, heavy exertion, and stressors induced by the dive profile are implicated in the increase in EVLW after diving.

## Methods

This prospective study consisted in an assessment of EVLW using ultrasonographic chest examinations in healthy volunteers who performed dives in the Mediterranean Sea.

### Population studied

Our study was an observational study performed during the daily activity of the French National Institute of Professional Diving. All divers participating in the teaching program (8 weeks) were included in the study from June 2012 to June 2014. While the frequency of the injury is rather low (around 1%), the increase in EVLW, suggesting minor pulmonary edema, is more frequent. For example, in the study of Castagna et al. [21], an EVLW accumulation was reported in 73% of divers. Consequently, the investigation of a population larger than 100 individuals was considered to be appropriate for the aim of our study.

Each subject passed a screening examination, which included a physical examination and a review of their medical history. The baseline investigations, including chest ultrasonography, were performed between the third and the eighth day after entering the diving center, in a period without diving activity. During the study, the subjects were investigated directly in the boat, inside a cabin with a controlled ambient temperature (25 °C), before and after the dives. The number of dives per subject varied according to the availability of the investigators and the weather conditions. In the case of several examinations for the same diver, the recordings were performed before and after each dive. The post-dive investigations, which included chest ultrasonography, were recorded between 5 and 60 min after the end of the dive. This delay was considered appropriate because Ljubkovic et al. [22] have reported that ULC disappeared within 2 or 3 h after surfacing. The diving parameters were also recorded (depth, duration at the bottom, duration of immersion, breathing apparatus, decompression stop duration, gas at depth and during decompression stops, diving suit type, and water temperature). The dive profile was recorded using a dive computer (Galileo Sol, Scubapro-Uwatec, Antibes, France). Furthermore, the occurrence of respiratory symptoms such as coughing, shortness of breath, expectorations or hemoptysis, during or after the dive was systematically researched. Lastly, the exercise intensity (using the Borg Rating of Perceived Exertion Scale—RPE) and cold perception (using a visual analog scale—VAS cold) experienced during the dive were quantified. The training program and the dive profiles were decided by the diving instructors. Consequently, the investigators were blinded to the dive profiles as well as to the stressors experienced by the divers during exercise at depth.

### Chest ultrasonography

The ultrasonographic examinations were carried out by experienced investigators using a commercially available Doppler echocardiograph (Esaote Mylab 30CV, Genoa, Italy) connected to a 2.5–3.5 MHz transducer array. The researchers were blinded to the clinical status as well as to the results of the questionnaire. Detection of ULC was performed using a recognized method [16, 19, 23] previously used for breath-hold divers [20]. A small number of ULC can be found in healthy subjects. In our work, according to past studies [24–26], an increase in ULC number of more than four artifacts when compared with the examination performed before the corresponding dive and a score greater than 5 was considered as a sign of increase in EVLW*.* The investigations were recorded on computers and were subsequently read by two independent observers.

### Statistical analysis

Continuous variables were expressed as mean ± standard deviation. All the statistical analyses were performed with R statistical software [27]. Each subject served as his own control. Two series of measurements were obtained: the first as a control before each dive and the second after the dive. We then searched for the factors associated to the increase in ULC number. First, linear relationship between our variables was described by using Spearman’s correlation.

Ultrasound examinations of the chest were performed between 2 and 12 times per subject, before and after diving. Number of diving instances per subject varied between one and six. Thus, we needed to take this source of randomness into account and assess the implied unbalance of our protocol. Because Ancova based on linear model with imbalanced data produced numerous difficulties [28], we used the most appropriate statistical tool, i.e., a mixed model with the following fixed effects: protocol factor, diving, individual factors such as age, weight, etc. and characteristics of the dive profile (e.g., duration of the dive, water temperature, etc.) and random effect: subjects.

Wald statistic was calculated and reported (*z* value) for each fixed effect. The *z* value is the Wald statistic for testing the hypothesis that the corresponding parameter (regression coefficient) is zero. Under the null hypothesis, it has an approximately N(0,1) distribution. P(> |z|) is the tail area in a two-tail test, i.e., the test within a two-sided outer hypothesis. Our dependent variable, a sign of increase in EVLW, is considered a rare event in a healthy population. Thus, we used a negative binomial model that takes into account overdispersion in the distribution of events.

We compared two models by Akaike information criterion (AIC) [29]: the first was composed by all the predictive variables with no interaction and a second with all the variables plus age crossed with the physical activity intensity level (Borg scale). Model with the lowest AIC was kept. The lme4 package [30] was used for these analyses. The incidence rate ratio was calculated with the exponent of the estimate when appropriate. To assess the stability of our results, we produced power calculation through simulation process: 100 new dependant variables were generated and 100 new mixed model calculations were computed thus proportion of success (*p* value equal or inferior to 0.05) defines as power.

## Results

### Population studied

One-hundred six divers (104 men, 2 women) were included in the study. Their mean age was 31 ± 7 years (from 19 to 58) with most divers (75%) between 27 (first quartile) and 35 years old (third quartile). The mean weight was 80 ± 12 kg, and the mean height was 178 ± 6 cm. At baseline (at admission into the diving institute), all divers had a number of ULC lower than 5: 76 divers had no ULC, 22 divers had 1 ULC, 4 divers had 2 ULC, 3 divers had 3 ULC, and 1 diver had 4 ULC. These ULC were mainly recorded on the lower intercostal spaces on the antero-lateral chest (parasternal, mid-clavicular, anterior axillary, and mid-axillary lines) on the right side (41% of cases) and on the left side (31%). ULC were more rarely recorded on the posterior chest (posterior axillary, scapular and paravertebral lines) on the right (16%) or on the left side (13%).

### Dives studied

In total, 263 dives (Table 1) were investigated (from one to six dives per subject). The water temperature was 16 ± 3 °C (range 10–23 °C). The divers breathed air at the bottom. They were submitted to a maximal partial pressure of oxygen (O_2_) from 0.38 to 1.22 ATA. The ascent rate was 9 to 15 msw.min^−1^, with a decompression stop at 3 and/or 6 msw and sometimes 9 msw, according to the recommendations of the decompression tables developed by the French Ministry of Labor in 1992 (MT92 table) [31]. During the decompression period at 3 and 6 msw, the divers breathed 100% O_2_; they breathed air at 9 msw. Forty-three successive dives were investigated.

**Table 1:** Characteristics of the dives studied

	HOOKAH	SCUBA
	Mean ± SD (min–max)	Mean ± SD (min–max)
Number of dives	202	61
Diving suit	Dry	Wet
Depth (msw)	30 ± 12 (8–48)	33 ± 11 (10–48)
Duration at the bottom (min)	43 ± 15 (9–77)	20 ± 7 (9–30)
Total duration of immersion (min)	64 ± 13 (18–113)	29 ± 7 (15–45)

*HOOKAH* dives performed with an open-circuit umbilical supplying compressed gas diving apparatus

*SCUBA* dives performed with a self-contained underwater breathing apparatus

### Examinations after the dives

Two divers reported one episode of breathlessness during the decompression stops. In the two cases, these troubles disappeared before the end of the water immersion stay and no respiratory trouble was recorded during the ultrasonographic examinations performed after the dive. In one diver, a significant increase in ULC number was recorded by chest ultrasonography, while in the other diver, no ULC was observed.

### Linear relationship between the variables

**Fig. 2:**
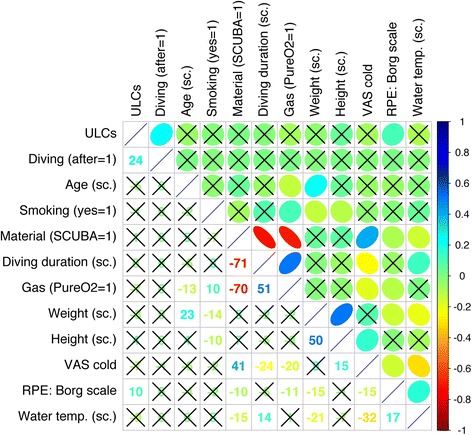
Correlogram reporting the relationship between the variables. Positive relationship is in color blue and negative relationship in red color. Absence of relation is in white. Non-significant correlations are crossed (*p* > .05). In the upper part, an ellipse represents the orientation and the strength of the relation, the narrower the ellipse is, the higher the strength is. In the lower part, the rounded Spearman rho is indicated. Order of the variables is determined by hierarchical clustering with the “complete” agglomeration method. “sc.” is an abbreviation for scaled (centered and scaled). *ULC* ultrasound lung comet, *SCUBA* self-contained underwater breathing apparatus, *Gas* gas during decompression stop, *O*_*2*_ oxygen, *VAS* visual analog scale, *RPE* rating of perceived exertion, *Temp*. temperature

To assess relationship between the studied variables, a correlation matrix was performed (Spearman’s rho). The correlation plot is reported in Fig. 2. High linear relationships were observed between diving duration (scaled) and material (SCUBA = 1, HOOKAH = 0), gas during decompression stops (O_2_ = 1, Air = 0) and material, gas during decompression stops and diving duration (scaled), height (scaled) and weight (scaled) VAS cold, and material. ULC number has linear relationship with only two variables: diving and RPE (Borg scale).

### Association and potential predictors

When comparing model with all variables plus interaction between age × Borg scale (RPE) and model with all variables without interaction, AIC was lower (923.89 vs. 925.64) for the model with no interaction. The simpler model (without interaction) was kept for the following analysis.

Results of the generalized linear mixed-effects model for the negative binomial family are reported in Table 2. Diving increased ULC number by 3.16 (IRR confidence interval, 2.01–4.57, *z* = 6.42, *p* = 1.37 × 10^−10^). The only other predictive variable of an increasing number of ULC was the exercise intensity (RPE) at depth (*z* = 3.99, *p* = 0.0000659). As seen in 3.4, diving has no significant linear relationship with other variables than ULCs, and RPE has weak linear relationship with material, gas during decompression stops, weight (scaled) VAS cold, and ULCs restraining confounding of a third variable.

**Table 2:** Estimate, standard error of estimate, confidence intervals of estimate obtained by bootstrap, *z* value, *p* value, and post hoc power are represented for fixed effects of generalized linear mixed-effects model for the negative binomial family

	Estimate	Std. error	2.5%	97.5%	*z* value	Pr(> |z|)	1-β
(Intercept)	−3.14	0.84	−4.11	−2.21	−3.74	0.000186*	1
Diving (after)	1.15	0.179	0.696	1.53	6.42	1.37 × 10^−10^*	1
Age (scaled)	−0.141	0.139	−0.414	0.126	−1.01	0.312	0.72
Smoking (yes)	−0.000678	0.279	−0.63	0.507	−0.0024	0.998	0.56
Material (SCUBA)	−0.0576	0.516	−0.898	0.602	−0.112	0.911	0.56
Total duration (scaled)	0.133	0.155	−0.167	0.397	0.857	0.392	0.69
Decompression Gas (O_2_)	−0.511	0.499	−1.3	0.171	−1.02	0.306	0.76
Weight (scaled)	−0.0603	0.143	−0.408	0.271	−0.421	0.674	0.55
Height (scaled)	0.175	0.151	−0.122	0.488	1.16	0.248	0.78
Coldness (VAS)	−0.0244	0.0617	−0.208	0.0899	−0.396	0.692	0.61
Water temperature (scaled)	−0.169	0.116	−0.376	0.0491	−1.46	0.145	0.8
RPE (Borg scale)	0.172	0.0431	0.106	0.229	3.99	6.59 × 10^−05^*	1

Diving (after) is the impact of the dive on the ULCs—examinations before and after the dive

*O*_*2*_ oxygen, *SCUBA* self-contained underwater breathing apparatus, *VAS* visual analog scale, *RPE* rating of perceived exertion

*Significant difference

## Discussion

The present study provides several interesting findings on the risk of increase in EVLW after diving in healthy subjects.

### Increase in extravascular lung water after diving

First, the occurrence of respiratory troubles in the context of well-controlled dives is rare. In the population studied, only 2 divers out of 106 reported one episode of breathlessness at the end of the dive during the decompression stop. These respiratory symptoms quickly disappeared, and the divers were asymptomatic when they were submitted to the investigations on board. In our work, chest ultrasonography was used to detect an increase in EVLW. One diver with breathlessness during the decompression stop had an elevated ULCs whereas the other had no ULC. Consequently, the respiratory troubles could not be related to minor pulmonary edema.

In the whole population, although a significant increase in ULC number was recorded after the dives, ULC number remained low. At the baseline examination, ULC number was lower than 5 in all volunteers. After the dives, few divers presented a ULC number greater than 5. According to the criteria chosen to define a significant increase in ULC number, only 8 divers out of 106 had ultrasonographic signs of increase in EVLW. This increase was recorded in 12 dives out of 263 investigated. Consequently, it can be concluded that an increase in EVLW rarely occurs after a well-controlled dive in healthy subjects. Given the infrequent occurrence of the increase in EVLW reported in our population of divers (104 men and only 2 women), our results should be interpreted with cautions.

### The contribution of exercise in the increase in extravascular lung water

The statistical analysis demonstrated that the intensity of exercise was the main contributor to the increase in EVLW. This result, recorded in our population of healthy subjects, agreed with physiological knowledge and previous case series of IPE. It is well recognized that strenuous exercise on land can induce PE in healthy subjects [32, 33]. During exercise on land, hemodynamic changes include an increase in both the cardiac output and pressure in pulmonary and systemic circulation. The increase in the pulmonary arterial pressure, leading to pulmonary capillary leakage and pulmonary capillary stress failure, is likely to be implicated in the pathophysiology of exercise pulmonary edema. In some individuals, such as subjects with left diastolic function impairment, an increase in both left atrial pressure and pulmonary capillary pressure can contribute to the pulmonary edema [34].

In water, supplementary cardio-respiratory strains are experienced. The high density of water in comparison to the air density, leads to an increase in ambient pressure. In SCUBA diving, it has been reported that the wet suit contributes to the increase in hydrostatic pressure [35]. This external hydrostatic pressure is transmitted to the smooth wall peripheral vessels reducing their compliance and leading to a blood mass transfer towards the central circulation [36–38]. In addition, the hydrostatic pressure also triggers a capillary shift of interstitial fluid into the blood compartment, which after some delay significantly increases the plasma volume [39–41]. The hemodynamic changes have been investigated at depth using underwater Doppler-echocardiography. A restrictive transmitral filling pattern, in favor of an impairment in left ventricular diastolic function leading to an increase in both left atrial pressure and pulmonary arterial wedge pressure, has been reported [42].

Furthermore, the myocardial wall stretch, due to the blood mass transfer, leads to the release of natriuretic peptides in the plasma, and is reported both after water immersion or SCUBA diving [22, 41, 43, 44]. Natriuretic peptides are recognized to increase endothelial permeability [45]. Lastly, the specific stressors induced by SCUBA breathing during exercise at depth might contribute to the development of PE through the increased workload of breathing, thus leading to great pleural pressure variations and subsequently to great variations in cardiac preload and after load [11]. Consequently, the cardiac, respiratory, and humoral alterations induced by water immersion could explain that exercising in water was the main contributor to the increase in EVLW in our study.

### The role of other environmental stressors

The other environmental stressors investigated in our work were not related to the increase in ULCs.

Cold exposure has been implicated in the pathogenesis of IPE [15]. Nevertheless, PE have also been described after diving in warm water [3, 20, 46]. In our study, the statistical analysis did not demonstrate a correlation between ULCs and water temperature or coldness. Consequently, cold exposure did not seem to be a major contributor to the increase in EVLW. Nevertheless, our study was carried out in the Mediterranean Sea and the water temperature ranged from 10 to 23 °C. One could object that the cold stressor was not sufficient enough. Further studies in fresh water would be interesting to better assess the contribution of cold in the pathogenesis of IPE.

No correlation between the increase in ULCs and the parameters of the dive profile was recorded in our work. The magnitude of the decompression stressor induced by the dive seemed unrelated to the increase in EVLW. This result agreed with the previous studies. In a study performed in 12 divers, Dujic et al. [47] did not observe any significant increase in ULCs after a no-decompression dive performed at 33 msw. In this work, a high bubble grade was recorded in the divers. In the study of Dujic et al. [47] and the present study, the profiles were controlled and the decompressions were performed according to the recommendations of the Norwegian and French diving tables, respectively. These results did not disagree with the fact that pulmonary edema can be induced by a major decompression stress such as pulmonary decompression sickness, i.e., “chokes” [48].

Lastly, hyperoxia might be implicated in the pathogenesis of IPE through an increase in the production of oxygen-free radicals. Indeed, it has been demonstrated that a long duration exposure to an increase in oxygen partial pressure was associated to endothelial damages in the pulmonary capillaries and alveoli, resulting in interstitial and alveolar edema [49]. In our work, for short duration dives (37 ± 16 min at the bottom) performed by healthy subjects, hyperoxia did not contribute to the increase in ULCs. This result could be related to the fact that the risk of IPE in military divers using oxygen-enriched gas mixtures through rebreathers did not seem to be higher than in swimmers or recreational divers [50, 51]. On the other hand, a protective effect of hyperoxia has been recently evoked. When compared with normoxia, hyperoxia counteracted the increase in both central venous pressure and mean arterial pulmonary pressure recorded at depth (4.7 ATA) during exercise [52]. In our work, no difference in ULCs was found after the dives between subjects breathing oxygen and subjects breathing air at the decompression stop. Nevertheless, our study was performed in a diving school of professional divers leading to the use of specific procedures and materials such as a HOOKAH. The decompression stops were most frequently performed using oxygen breathing (88% of cases). A few SCUBA dives, breathing air for the whole duration of the dive, were investigated (31 dives). Although these SCUBA divers did not develop more numerous ULC after diving than others (232 dives), the small sample studied led to an impairment of the power of the statistical analysis. Furthermore, if the protective effect of hyperoxia is real, the procedure used in our diving center could explain the scarcity of the increase in EVLW recorded after the dives, when compared with the recent study of Castagna et al. [21]. Consequently, it seems important to perform further studies on the potential protector effect of hyperoxia against IPE, in particular among SCUBA divers.

## Conclusions

According to the results of our prospective study, the main contributor to the increase in EVLW in healthy divers is exercise intensity. The other environmental stressors investigated such as the temperature of the water, the dive profile, and the hyperoxic exposure were not significantly related to the increase in ULC number assessed by chest ultrasonography. These findings are important to improve the prevention of IPE in divers. Indeed, before dive training, it is important to assess the aerobic fitness of the divers and subsequently adapt the exercise intensity experienced during the dive to the aerobic fitness level of the individual diver.

## Abbreviations

AIC: Akaike information criterion
ATA: Atmosphere absolute
EVLW: Extra vascular lung water
HOOKAH: Open-circuit umbilical supplying compressed gas
IPE: Immersion pulmonary edema
msw: meters of sea water
O_2_: Oxygen
PE: Pulmonary edema
SCUBA: Self-contained underwater breathing apparatus
ULC: ultrasound lung comets
ULCs: ULC score

## References

[1] Wilmshurst PT, Nuri M, Crowther A (1981). Forearm vascular responses in subjects who develop recurrent pulmonary edema when SCUBA diving : a new syndrome. Br Heart J.

[2] Boussuges A, Pinet C, Thomas P (1999). Haemoptysis after breath-hold diving. Eur Respir J.

[3] Hampson NB, Dunford RG (1997). Pulmonary oedema of SCUBA divers. Undersea Hyperb Med.

[4] Koehle MS, Lepawsky M, McKenzie DC (2005). Pulmonary oedema of immersion. Sports Med.

[5] Pons M, Blickenstorfer D, Oechslin E (1995). Pulmonary oedema in healthy persons during scuba-diving and swimming. Eur Respir J.

[6] Cochard G, Arvieux J, Lacour JM (2005). Pulmonary edema in scuba divers: recurrence and fatal outcome. Undersea Hyperb Med.

[7] Dwyer N, Smart D, Reid DW (2007). SCUBA diving, swimming and pulmonary oedema. Intern Med J.

[8] Gempp E, Boussuges A, Poyet R (2009). Acute coronary event revealed by diving-related pulmonary edema. Ann Cardiol Angeiol.

[9] Kenealy H, Whyte K (2008). Diving-related pulmonary oedema as an unusual presentation of alcoholic cardiomyopathy. Diving Hyperb Med.

[10] Ludwig BB, Mahon RT, Schwartzman EL (2006). Cardiopulmonary function after recovery from swimming-induced pulmonary edema. Clin J Sports Med.

[11] Slade JB, Hattori T, Ray CS (2001). Pulmonary edema associated with scuba diving: case reports and review. Chest.

[12] Peacher DF, Martina SD, Otteni CE (2015). Immersion pulmonary edema and comorbidities: case series and updated review. Med Sci Sports Exerc.

[13] Coulange M, Rossi P, Gargne O (2010). Pulmonary oedema in healthy SCUBA divers: new physiopathological pathways. Clin Physiol Funct Imaging.

[14] Miller CC, Calder-Becker K, Modave F (2010). Swimming-induced pulmonary edema in triathlete. Am J Emerg Med.

[15] Wilmshurst PT, Nuri M, Crowther A (1989). Cold-induced pulmonary edema in scuba divers and swimmers and subsequent development of hypertension. Lancet.

[16] Lichtenstein D, Mézière G, Biderman P (1997). The comet-tail artifact. An ultrasound sign of alveolar-interstitial syndrome. Am J Respir Crit Care Med.

[17] Pratali L, Cavana M, Sicari R (2010). Frequent subclinical high-altitude pulmonary edema detected by chest sonography as ultrasound lung comets in recreational climbers. Crit Care Med.

[18] Pingitore A, Garbella E, Piaggi P (2011). Early subclinical increase in pulmonary water content in athletes performing sustained heavy exercise at sea level: ultrasound lung comet-tail evidence. Am J Physiol Heart Circ Physiol.

[19] Frassi F, Pingitore A, Cialoni D (2008). Chest sonography detects lung water accumulation in healthy elite apnea divers. J Am Soc Echocardiogr.

[20] Boussuges A, Coulange M, Bessereau J (2011). Ultrasound lung comets induced by repeated breath-hold diving: a study in underwater fishermen. Scand J Med Sci Sports.

[21] Castagna O, Gempp E, Poyet R (2017). Cardiovascular mechanisms of extravascular lung water accumulation in divers. Am J Cardiol.

[22] Ljubkovic M, Gaustad SE, Marinovic J (2010). Ultrasonic evidence of acute interstitial lung edema after SCUBA diving is resolved within 2-3h. Respir Physiol Neurobiol.

[23] Agricola E, Bove T, Oppizzi M (2005). Ultrasound comet-tail images: a marker of pulmonary edema. Chest.

[24] Picano E, Frassi F, Agricola E (2006). Ultrasound lung comets: a clinically useful sign of extravascular lung water. J Am Soc Echocardiogr.

[25] Gargani L (2011). Lung ultrasound: a new tool for the cardiologist. Cardiovasc Ultrasound.

[26] Lambrechts K, Germonpré P, Charbel B (2011). Ultrasound lung “comets” increase after breath-hold diving. Eur J Appl Physiol.

[27] R Core Team. R: A language and environment for statistical computing. Vienna, Austria; 2015. URL https://www.r-project.org/. 2015-03-09.

[28] Shaw RG, Mitchell-Olds T (1993). ANOVA for unbalanced data: an overview. Ecology.

[29] Akaike H (1974). A new look at the statistical model identification. IEEE Trans Autom Control.

[30] Bates D, Maechler M, Bolker B (2015). Fitting linear mixed-effects models using lme4. J Stat Softw.

[31] Imbert JP, Broussolle B, Méliet JL (2006). Calcul des tables du Ministère du travail MT92. Physiologie et Médecine de la Plongée.

[32] Hopkins SR, Schoene RB, Henderson WR (1997). Intense exercise impairs the integrity of the pulmonary blood-gas barrier in elite athletes. Am J Respir Crit Care Med.

[33] Zavorsky GS (2007). Evidence of pulmonary edema triggered by exercise in healthy humans and detected with various imaging techniques. Acta Physiol (Oxf).

[34] Agricola E, Picano E, Oppizzi M (2006). Assessment of stress-induced pulmonary edema by chest ultrasound during exercise echocardiography and its correlation with left ventricular function. J Am Soc Echocardiogr.

[35] Castagna O, Blatteau JE, Vallee N (2013). The underestimated compression effect of neoprene wetsuit on divers hydromineral homeostasis. Int J Sports Med.

[36] Arborelius M, Balldin UI, Lilja B (1972). Hemodynamic changes in man during immersion with the head above water. Aerosp Med.

[37] Gabrielsen A, Johansen LB, Norsk P (1993). Central cardiovascular pressures during graded water immersion in humans. J Appl Physiol.

[38] Risch WD, Koubenec HJ, Beckmann U (1978). The effect of graded immersion on heart volume, central venous pressure, pulmonary blood distribution, and heart rate in man. Pfluggers Arch.

[39] Khosla SS, DuBois AB (1981). Osmoregulation and interstitial fluid pressure changes in humans during water immersion. J Appl Physiol Respir Environ Exerc Physiol.

[40] Miki K, Hajduczok G, Hong SK (1986). Plasma volume changes during head-out water immersion in conscious dogs. Am J Phys.

[41] Ayme K, Rossi P, Gavarry O (2015). Cardiorespiratory alterations induced by low-intensity exercise performed in water or on land. Appl Physiol Nutr Metab.

[42] Marabotti C, Cialoni D, Pingitore A (2017). Environment-induced pulmonary oedema in healthy individuals. Lancet Respir Med.

[43] Norsk P, Bonde-Petersen F, Christensen NJ (1990). Catecholamines, circulation, and the kidney during water immersion in humans. J Appl Physiol.

[44] Passino C, Franzino E, Giannoni A (2011). B-type natriuretic peptide secretion following scuba diving. Biomark Med.

[45] Curry FR (2005). Atrial natriuretic peptide: an essential physiological regulator of transvascular fluid, protein transport, and plasma volume. J Clin Invest.

[46] Gnadinger CA, Colwell CB, Knaut AL (2001). Scuba diving-induced pulmonary edema in a swimming pool. J Emerg Med.

[47] Dujic Z, Marinovic J, Obad A (2011). A no-decompression air dive and ultrasound lung comets. Aviat Space Environ Med.

[48] Kondo Y, Shiohira S, Kamizato K (2012). Vascular hyperpermeability in pulmonary decompression illness: 'the chokes. Emerg Med Australas.

[49] Crapo JD (1986). Morphologic changes in pulmonary oxygen toxicity. Annu Rev Physiol.

[50] Arieli R, Shochat T, Adir Y (2006). CNS toxicity in closed-circuit oxygen diving: symptoms reported from 2527 dives. Aviat Space Environ Med.

[51] Gempp E, Louge P, Blatteau JE (2011). Descriptive epidemiology of 153 diving injuries with rebreathers among French military divers from 1979 to 2009. Mil Med.

[52] Peacher DF, Pecorella SR, Freiberger JJ (2010). Effects of hyperoxia on ventilation and pulmonary hemodynamics during immersed prone exercise at 4.7 ATA: possible implications for immersion pulmonary edema. J Appl Physiol.

